# Nrf2 Expression Is Regulated by Epigenetic Mechanisms in Prostate Cancer of TRAMP Mice

**DOI:** 10.1371/journal.pone.0008579

**Published:** 2010-01-05

**Authors:** Siwang Yu, Tin Oo Khor, Ka-Lung Cheung, Wenge Li, Tien-Yuan Wu, Ying Huang, Barbara A. Foster, Yuet Wai Kan, Ah-Ng Kong

**Affiliations:** 1 Department of Pharmaceutics, Ernest Mario School of Pharmacy, Rutgers, The State University of New Jersey, Piscataway, New Jersey, United States of America; 2 Department of Pharmacology and Therapeutics, Roswell Park Cancer Institute, Buffalo, New York, United States of America; 3 Cardiovascular Research Institute and Departments of Laboratory Medicine and Medicine, University of California San Francisco, San Francisco, California, United States of America; University of Minnesota, United States of America

## Abstract

Nuclear factor-erythroid 2 p45-related factor 2 (Nrf2) is a transcription factor which regulates the expression of many cytoprotective genes. In the present study, we found that the expression of Nrf2 was suppressed in prostate tumor of the Transgenic Adenocarcinoma of Mouse Prostate (TRAMP) mice. Similarly, the expression of Nrf2 and the induction of NQO1 were also substantially suppressed in tumorigenic TRAMP C1 cells but not in non-tumorigenic TRAMP C3 cells. Examination of the promoter region of the mouse Nrf2 gene identified a CpG island, which was methylated at specific CpG sites in prostate TRAMP tumor and in TRAMP C1 cells but not in normal prostate or TRAMP C3 cells, as shown by bisulfite genomic sequencing. Reporter assays indicated that methylation of these CpG sites dramatically inhibited the transcriptional activity of the Nrf2 promoter. Chromatin immunopreceipitation (ChIP) assays revealed increased binding of the methyl-CpG-binding protein 2 (MBD2) and trimethyl-histone H3 (Lys9) proteins to these CpG sites in the TRAMP C1 cells as compared to TRAMP C3 cells. In contrast, the binding of RNA Pol II and acetylated histone H3 to the Nrf2 promoter was decreased. Furthermore, treatment of TRAMP C1 cells with DNA methyltransferase (DNMT) inhibitor 5-aza-2′-deoxycytidine (5-aza) and histone deacetylase (HDAC) inhibitor trichostatin A (TSA) restored the expression of Nrf2 as well as the induction of NQO1 in TRAMP C1 cells. Taken together, these results indicate that the expression of Nrf2 is suppressed epigenetically by promoter methylation associated with MBD2 and histone modifications in the prostate tumor of TRAMP mice. Our present findings reveal a novel mechanism by which Nrf2 expression is suppressed in TRAMP prostate tumor, shed new light on the role of Nrf2 in carcinogenesis and provide potential new directions for the detection and prevention of prostate cancer.

## Introduction

Prostate cancer, as well as many other age-related cancers, is characterized by increased intracellular oxidative stress [1], [2]. Chronic oxidative stress and its associated pathological conditions including inflammation and metabolic disorders have been postulated to drive somatic mutations and neoplastic transformation, thus could play an important role in the development and progression of prostate cancer [3]. Increased oxidative stress or reactive oxygen species (ROS) levels could be a consequence of increased ROS generation and/or decreased antioxidant capacities and or ROS detoxification. Recently the impaired antioxidant defense system in carcinogenesis of prostate cancer has been gaining increased attentions.

The cellular antioxidant defense system comprises a battery of antioxidant/detoxifying enzymes and proteins such as superoxide dismutase (SOD), catalase, hemeoxgenase (HO), UDP-glucuronosyltransferases (UGT), glutathione peroxidase (GPx), glutathione S-transferases (GST), and NAD(P)H:quinone oxidoreductase (NQO) [4]. Down-regulation of GST by DNA methylation appears to be quite common in human prostate cancer that it has been developed as a diagnostic marker [5]. The expression and the activities of SOD, catalase and GPx have been reported to be decreased in prostate carcinoma tissues as well as in plasma and erythrocytes [6]–[8]. Recent studies from our laboratory and others have found that the expression levels of SOD, UGT1A1, NQO1 and several GST family genes were significantly suppressed in prostate tumors in Transgenic Adenocarcinoma of Mouse Prostate (TRAMP) mice [9]–[11]. Although the down-regulation of GST enzymes in human prostate cancer have been linked to the promoter hypermethylation of GST genes [5], [12]; promoter DNA hypermethylation does not appear to cause GST gene repression in TRAMP tumors [9]. Instead, down-regulation of nuclear factor-erythroid 2p45 (NF-E2)-related factor 2 (Nrf2), a key regulator of cellular antioxidant enzymes, may be responsible for the transcriptional suppression of GSTs and other phase II detoxifying enzyme genes [11].

Nrf2 is a helix–loop–helix basic leucine zipper transcription factor that regulates the expression of many phase II detoxifying and antioxidant enzymes via its binding to the antioxidant-response element (ARE) in the promoter region [4]. Knockout of Nrf2 in mice substantially abrogated the inducible expression of ARE-mediated detoxifying and antioxidant enzymes, and rendered these mice highly susceptible to carcinogens and/or oxidative damages [13], [14]. In these context, previously we have found that the protein expression levels of Nrf2 and Nrf2-target gene heme-oxygenase-1 (HO-1) were attenuated in the skin tumors of a mouse skin carcinogenesis model [15]. Similarly, the expression of Nrf2 as well as its downstream target genes such as UGT1A1, GSTM1 and NQO1 were found to be gradually down-regulated in prostate tumors with the progression of prostate tumorigenesis in TRAMP mice [10]. Frolich et al. recently reported the expression of Nrf2 and GST mu family genes were significantly decreased in TRAMP prostate tumor, and linked this phenomenon to increased oxidative stress and DNA damage in prostate cancer. Meta-analysis of tissue expression profiling data from Oncomine database (www.oncomine.org) suggested that the expressions of Nrf2 and several GST mu genes are also gradually down-regulated in human prostate cancers [11].

The transcription of Nrf2 has recently been shown to be regulated by the aryl hydrocarbon receptor (AhR) and Nrf2 itself [16], [17]. However, the currently accepted paradigm of Nrf2 regulation appears to be mainly achieved via post-translational mechanisms. As such, Nrf2 is functionally suppressed by the Kelch-like Erythroid-cell-derived protein with CNC homology (ECH)-Associated Protein 1 (Keap1), which binds to and sequesters Nrf2 in the cytoplasm leading to the degradation of Nrf2, and thus prevents Nrf2 nuclear translocation and transactivation of its target genes [18]. Upon challenges by oxidative or electrophilic stresses that could involve potential modification of critical cysteine residues in Keap1 and or Nrf2 itself coupled with phosphorylation by kinases, Nrf2 is released from Keap-1, translocates into the nucleus, dimerizes with small Maf proteins, binds to ARE and transcriptionally activates Nrf2-ARE target genes [4]. To date, it is not clear as to how the expression of Nrf2 in human prostate cancer or in TRAMP mouse tumor is suppressed.

Epigenetic or epigenomic mechanisms, particularly DNA methylation, have been frequently implicated in the alterations of gene expression in prostate cancer [19], [20]. Coordinated hypermethylation of APC and GSTP1 in early carcinogenesis has been utilized as potential diagnostic markers to detect prostate cancer [21]. In addition, alteration of DNA methylation profiles has been linked with cancer progression [20]. DNA methylation, coupled with histone modifications, would affect the interactions of the promoters of critical genes with transcriptional corepressors and coactivators leading to changes in gene expressions, which could be one of the driving forces for prostate carcinogenesis. Silencing of multiple genes by DNA methylation has been reported in TRAMP prostate tumors and cell lines derived from TRAMP prostate tumors [22]–[24]. Inhibition of DNA methyltransferase activity by 5-aza-2′-deoxycytidine (5-aza) has been shown to prevent prostate tumorigenesis in TRAMP mice [25]. In addition, the expression of Keap1 has been reported to be regulated by DNA methylation in lung cancer [26]. In the present study, we first identified a CpG island in the upstream 5′-flanking region of the murine Nrf2 gene followed by interrogation of the DNA methylation status of the whole CpG island via bisulfite genomic sequencing. We found that certain CpG sites in the distal region of the CpG island were hypermethylated in the TRAMP tumor tissues as well as in tumorigenic TRAMP-C1 and -C2 cells, but not in normal prostatic tissue and non-tumorigenic TRAMP-C3 cells. Utilizing methylated reporter assay, chromatin immunoprecipitation (ChIP) assay and treatments with trichostatin A (TSA)/5-aza, we provided compelling evidence that the expression of Nrf2 is epigenetically regulated during the development of prostate tumors in TRAMP mice. Nrf2 expression was suppressed by methylation of certain CpG sites and this was accompanied by the recruitment of MBD2 and trimethyl-histone H3 (Lys9) to the Nrf2 gene promoter in prostate tumor of TRAMP mice.

## Results

### Hypermethylation of Specific CpG Sites in the CpG Island in Murine Nrf2 Gene

**Figure 1:**
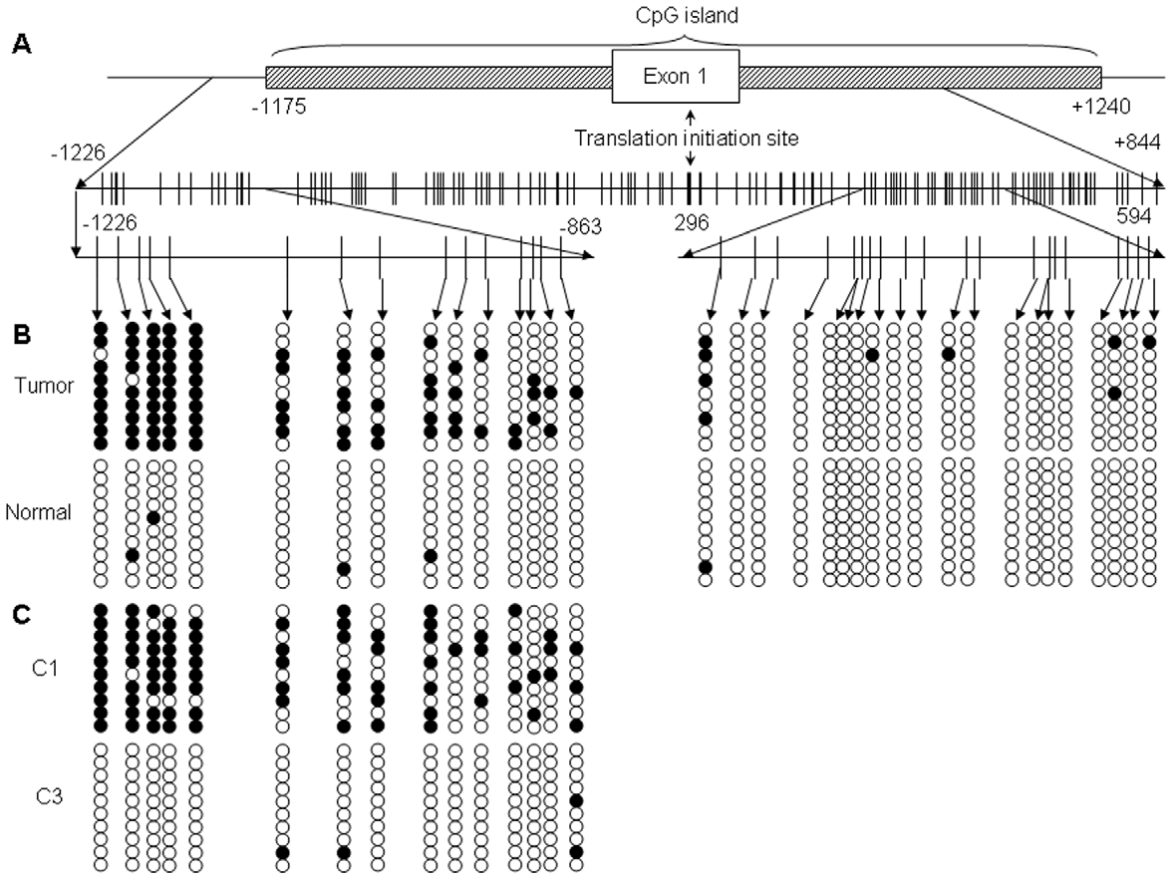
Hypermethylation of Nrf2 promoter in TRAMP prostate was correlated with tumorigenesis. (**A**) A CpG island was identified in the 5′-flanking region of mouse Nrf2 gene, spanning from position −1175 to +1240 with the translation initiation site set as position 1. The sequences covered by bisulfite genomic sequencing (−1226 to +844) and contain methylated CpGs are schematically presented with CpG sites indicated by vertical lines. (**B**) The methylation patterns and extents of CpG sites in the promoter of Nrf2 gene in TRAMP prostate tumor and apparently normal prostate were determined by bisulfite genomic sequencing as described in Material & Methods. Black dots indicate methylated CpGs and open circles indicate non-methylated CpGs. (**C**) The methylation patterns and extents of CpG sites in the promoter of Nrf2 gene in TRAMP C1 and C3 cells were determined. The CpGs in the sequence between +296 to +594 are not displayed because methylation is insignificant.

The genomic sequence of Nrf2 gene (NC_000068.6: 75544698-75513576 Mus musculus chromosome 2, reference assembly (C57BL/6J), including 2 kb of its 5′-upstream sequence) was analyzed for CpG island using CpG island Finder (http://people.usd.edu/~sye/cpgisland/CpGIF.htm). Since several mRNA variants with different transcription start sites (TSS) have been reported in the literature [16], [27], [28], therefore in the present study we define the translation initiation site (TIS) as position 1 to avoid any possible confusion. A CpG island was identified between -1175 and +1240, with a GC content of 61.53%, CpG observed/expected ratio of 0.66 and a total of 150 CpGs. The CpG island includes the murine Nrf2 promoter, the first exon and part of the first intron (Figure 1A). Similar results were obtained when using other criteria and algorithm (http://www.uscnorris.com/cpgislands2/cpg.aspx, data not shown). 10 sets of bisulfite genomic sequencing (BGS) primers were designed using the BiSearch web server (http://bisearch.enzim.hu/, [29]) to cover the 5′-flanking region spanning from −1226 to +844 of the murine Nrf2 gene (Table S1). Bisulfite-converted genomic DNA derived from 12 palpable prostate tumors of 24 weeks old TRAMP mice and 10 apparently normal prostate tissues of C57BL/6J mice was used as templates. As shown in Figure 1B, although the majority of the CpG island is barely methylated, the first 5 CpG sites were found to be hypermethylated (96%) in prostate tumors compared to apparently normal prostate tissues (4%). Representative sequencing chromatographs showing the first 5 CpGs in prostate tumor and normal prostate are shown in Figure S1. The following 10 CpGs that are separated by two CpG-free gaps (−1131 to −1060 and −886 to −798) also displayed differential methylation status in tumors (34%) compared to normal tissues (2%). In addition, another region located in the first intron appears to be sparsely CpG-methylated in prostate tumor (4.5%).

TRAMP C1, C2 and C3 cell lines were originated from a heterogeneous tumor of 32-week TRAMP mouse [30]. While TRAMP C1 and C2 cells are tumorigenic when grafted into syngenic C57BL/6J hosts, TRAMP C3 cells are not. Genomic DNA from C1 and C3 cells was bisulfite-sequenced as above to detect the methylation status of the CpG island in Nrf2 gene. Surprisingly, the methylated CpG “hot spots” as found above in the TRAMP prostate tumor were also methylated in tumorigenic C1 cells but not in non-tumorigenic TRAMP C3 cells (Figure 1C).

### Methylation of the First 5 CpGs Significantly Suppressed the Transcriptional Activity of Mouse Nrf2 Promoter

**Figure 2:**
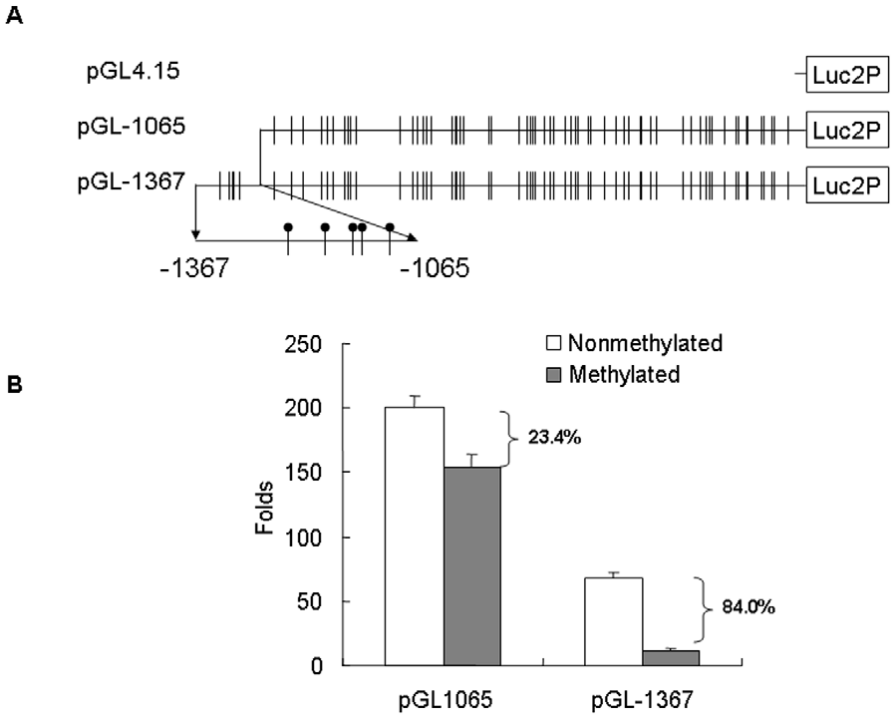
Methylation of the first 5 CpGs inhibited the transcriptional activity of Nrf2 promoter. (**A**) The construction of luciferase reporters is schematically presented. Nrf2 promoters with (−1367–1) or without (−1065–1) the extra sequence containing the first 5 CpGs were amplified from mouse genomic DNA and inserted into pGL 4.15 vector. The resulted reporters were designated as pGL-1367 and pGL-1065, respectively. (**B**) pGL-1367 or pGL-1065 reporters, either methylated by CpG methyltransferase or not, were co-transfected with pGL 4.75 vector which contains a Renilla reniformis luciferase gene driven by CMV promoter into TRAMP C1 cells, and the luciferase activities were measured after 24 hrs. The transcriptional activities of each constructs were calculated by normalizing the firefly luciferase activities with corresponding Renilla luciferase activities, and are represented as folds of induction compared with the activity of empty pGL 4.15 vector. The values are mean±SD of four separate samples.

To investigate the functional role of methylation of specific CpG sites, particularly the first 5 CpGs in the CpG island, luciferase reporters driven by the Nrf2 promoter with or without the first 5 CpGs (designated as −1367 and −1065, respectively) were constructed as described in Materials and Methods (Figure 2A). The plasmids were methylated using M. sssI CpG methyltransferase *in vitro* and the methylation status was confirmed by HpaI/HhaII digestion (Figure S2). As shown in Fig. 2B, the −1065 Nrf2 promoter, which had been reported previously [17], [28], substantially increased the luciferase activity to about 200 folds. In contrast, the −1367 Nrf2 promoter showed a less potent transcriptional activity (∼67 folds). Importantly, *in vitro* CpG-methylation of the reporter construct resulted in a dramatic decrease of the transcriptional activity of the −1367 Nrf2 promoter (84% decrease); while, the activity of the −1065 promoter was decreased by only 23.4% by CpG-methylation. Similar results were obtained in Hep G2 hepatoma cells and human embryonic kidney HEK 293 cells (Figure S3).

### Hypermethylated CpG Island Was Associated with Methyl-CpG-Binding Domain (MBD2) and Histone Modifications, Which Is Reversible by 5-aza/TSA Treatment

**Figure 3:**
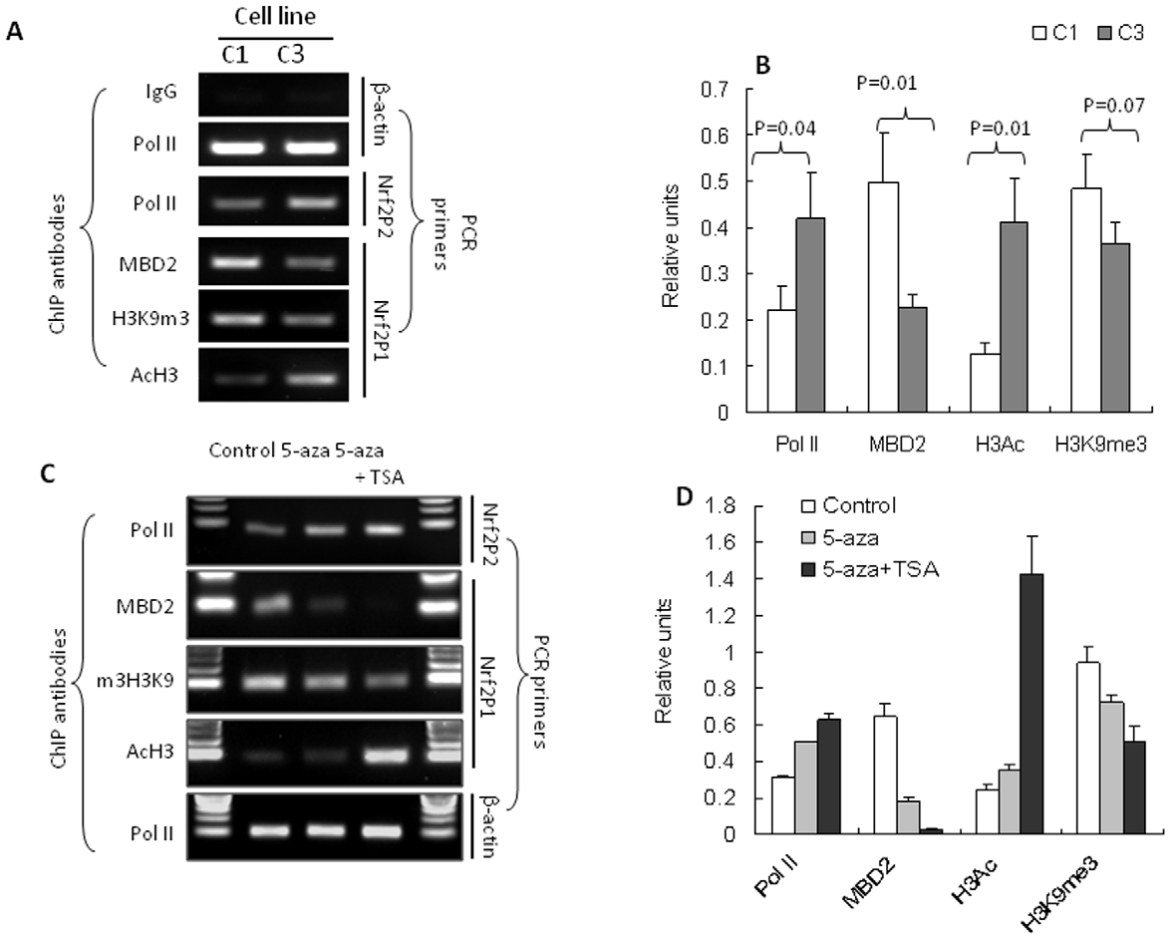
Hypermethylated CpG island was associated with MBD2 binding and histone modifications and 5-aza/TSA treatment reversed the association. (**A**) ChIP assay was performed to detect the binding of indicated proteins to specific regions of Nrf2 gene cross-linked and immunoprecipitated from TRAMP C1 and C3 cells. The results from 3 independent experiments were quantified by densitometry as shown in (**B**). (**C**) TRAMP C1 cells were treated with vehicle, 5-aza or 5-aza+TSA as described, then the cells were subjected to ChIP assay. The results from 2 independent experiments were quantified by densitometry as shown in (**D**). ChIP assays were performed as described in Material & Methods using antibodies against Pol II, MBD2, H3K9m3 and AcH3. 3 sets of ChIP primers were used (Table S2), with Nrf2P1 covers the first 5 CpGs (−1190 to −1092) in the CpG island and Nrf2P2 covers a region close to TSS (−62 to +20). Nonspecific IgG was employed as a negative control and binding of Pol II to β-actin promoter was used to verify the efficiency of ChIP assay. The experiments were repeated at least twice with similar results.

The repression of gene expression by CpG methylation is generally achieved by MBD proteins that bind to methylated DNA leading to recruitment of chromatin remodeling and transcriptional repressor complexes [31]. In the current study, ChIP assays were employed to examine the proteins that could be potentially associated with Nrf2 promoter in TRAMP C1 and C3 cells. Based on the methylation status of Nrf2 promoter, primer sets were designed to cover the first 5 CpGs and the TSS to detect the association of specified DNA-binding proteins as well as RNA polymerase II (Pol II). The specificity of ChIP assays were verified by nonspecific IgG which did not pull down anything. As a positive control, β-actin promoter is equally associated with Pol II in C1 and C3 (Figure 3A). The binding of Pol II to the Nrf2 gene TSS was significantly decreased in C1 cells as compared to in C3 cells, indicating a suppressed transcription of Nrf2 in C1 cells (Figure 3A & B). In agreement with this observation, the binding of MBD2 and tri-methylated histone 3-lys9, (H3K9me3) to the methylated CpGs in Nrf2 promoter was higher in C1 cells than in C3 cells, whereas the association of acetylated histone 3 (H3Ac) displayed a reversed pattern (Figure 3A & B). Methyl CpG binding protein 2 (MeCP2) and mammalian Sin3A (mSin3A) were also tested for their binding with this Nrf2 promoter; however no detectable binding was observed (data not shown).

The methylation status of DNA is regulated by DNA methyl-transferases (DNMTs) and 5-aza is a DNMT inhibitor, is often used to examine the effects of DNA methylation [32]. As shown in Figure 3C & D, 5-aza treatment of C1 cells decreases the binding of MBD2 and H3K9me3 to the Nrf2 promoter, while the H3Ac exhibits no observable effect. However, combined treatment of C1 cells with 5-aza and a histone deactylase (HDAC) inhibitor, TSA, substantially increases the binding of H3Ac to the Nrf2 promoter, and further decreases the bindings of MBD2 and H3K9me3 to the Nrf2 promoter. Consistent with the above results, the recruitment of Pol II to the Nrf2 promoter also increases correspondingly, whereas Pol II's binding to β-actin promoter was not changed (Figure 3C).

### The Transcriptional Induction of Nrf2 and NQO-1 in TRAMP Cell Lines Was Correlated with CpG Islands Methylation Status and Could Be Restored by 5-aza/TSA Treatment

**Figure 4:**
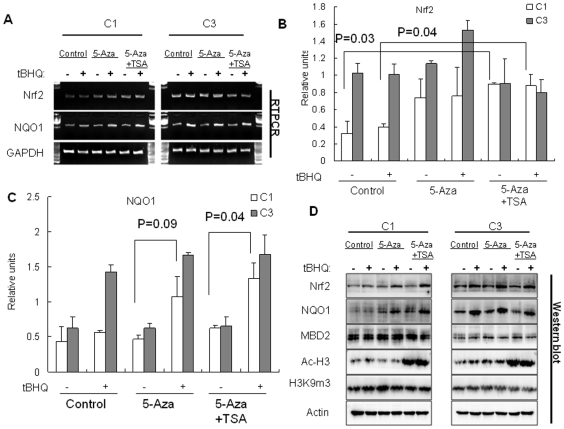
The expression of Nrf2 and NQO-1 in TRAMP cell lines was correlated with CpG islands methylation status and could be restored by 5-aza/TSA treatment. TRAMP C1 and C3 cells were treated with 2 µM 5-aza, 200 nM TSA, or 1 µM 5-aza plus 100 nM TSA for 60 hrs or 48 hrs followed by incubation in the presence of 5 µM tBHQ for further 12 hrs. After treatments, the cells were harvested for total protein or RNA extractions. (**A**) the mRNA levels of Nrf2 and NQO1 were determined by RT-PCR, with GAPDH serving as internal control, and the results from 3 independent experiments were quantified by densitometry and the results are shown in **B** and **C**. (**D**) the protein levels of Nrf2, NQO1, MBD2, H3K9me3 and H3Ac were determined by Western blotting, with actin as a loading control. Each experiment was repeated at least twice with similar results.

We and others have previously reported that the expression of Nrf2 and its target genes is suppressed in TRAMP prostate tumors [10], [11]. Marvis et al., had reported that the expression of GST family genes was suppressed in TRAMP C2 cells and 5-aza and TSA treatment could restore the expression [9]. Here we examined the expression of Nrf2 and one of its downstream target genes NQO1 in TRAMP C1 and C3 cells. As shown in Figure 4A, the mRNA level of Nrf2 is significantly lower in C1 cells than in C3 cells. The expression of NQO1 was readily induced by tBHQ, a classic Nrf2 agonist, in C3 cells, while such induction was blunted in C1 cells (Figure 4A & C). Treatment of C1 cells with 5-aza and TSA modestly restored Nrf2 expression and significantly enhanced the induction of NQO1 by tBHQ. However, the treatment of C3 cells under the same conditions exhibited no effect on the expression of Nrf2 or NQO1 (Figure 4A, B & C). Western blotting was performed to examine the protein expression levels of Nrf2, NQO1, MBD2, H3Ac and H3K9me3 (Figure 4D). The protein levels of Nrf2 and NQO1 were lower in C1 cells than in C3 cells, and 5-aza and TSA treatment enhanced the induction of NQO1 protein by tBHQ. Although 5-aza and TSA treatment did not increase the basal level of Nrf2 protein the treatment significantly augmented tBHQ-induced Nrf2 protein expression. TSA treatment strongly increased acetylated histone 3 protein while had no effect on histone 3 methylation status. Interestingly, although the protein level of MBD2 was not affected by 5-aza and TSA treatment, it was much higher in C1 cells than in C3 cells (Figure 4D).

## Discussion

Previous findings from several laboratories including our laboratory have demonstrated that Nrf2 plays an essential role in the development of various cancers [33]. Nrf2 regulates the expression of antioxidant and phase II detoxifying enzymes including NQO1, HO-1 and GST [33]. Therefore, the control of transcriptional activation of Nrf2 and Nrf2-target genes would appear to be an important homeostatic mechanism that protect cellular injuries or damages resulted from oxidative stress [33]. Nrf2 deficiency could lead to defect in the cellular defense system against oxidative stress, potentially resulting in cancer initiation, promotion and progression [34]. The repressed expression of antioxidant and detoxifying enzymes such as GSTP1 in prostate cancer has extensively been studied [2], [5]. However the role of Nrf2 in prostate cancer have not received enough attention until recently [10], [11].

Frolich et al. reported that the down-regulation of Nrf2 appears to be responsible for the reduced GST expression, elevated oxidative stress and DNA damage in prostate tumorigenesis in TRAMP mice [11]. We have recently found that the expression of Nrf2 as well as Nrf2-target genes is gradually down-regulated during the progression of prostate cancer in TRAMP mice [10]. Previous analysis of the online human prostate gene expression data sets demonstrated that the expression of Nrf2 and GST [11] as well as NQO1 was gradually decrease during human prostate carcinogenesis (Figure S4). Furthermore, it has been reported that several GST genes are down-regulated in primary but not in metastatic TRAMP tumors [9]. In current study, we found that the expression of Nrf2 and the induction of NQO1 was compromised in tumorigenic TRAMP C1 cells but not in non-tumorigenic TRAMP C3 cells (Figure 4A). This suppression of Nrf2 expression and Nrf2-target gene NQO1 in both of these TRAMP cell lines would exclude the possibility that Nrf2 expression would be affected by the SV40 transgene, since these TRAMP cell lines do not express the SV40 transgene [30].

DNA methylation has been implicated in the silencing of the GSTP1 gene in human prostate cancer, and similarly DNA-methylation silencing of several other genes are also implicated in TRAMP prostate tumor [5], [23], [24]. However, interestingly MassARRAY Quantitative DNA Methylation Analyses (MAQMA) analysis of the 5′ region of several GST genes displayed no significant differences between normal prostatic epithelial cells and prostate tumor from the TRAMP mice [9]. Recently, several reports show that in both TRAMP and Rb−/− prostate tumors, an Rb/E2F-dependent increase of DNMT1 expression and methylation activity [25], [35]. Hypermethylation Nrf2 promoter was ruled out using MSP and that 5-aza treatment had no effect on Nrf2 expression (with data not shown) [11]. We analyzed the 5′-flanking region of Nrf2 gene and identified a CpG island that extends to position -1175 (Figure 1A). Using bisulfite sequencing, which would be more specific in identifying CpG methylation and would reveal more details about DNA methylation than MSP, we found that the first 5 CpGs in the CpG island are hypermethylated in TRAMP prostate tumors and in the tumorigenic TRAMP C1 cells but not in normal prostate tissues and non-tumorigenic TRAMP C3 cells (Figure 1B). Remarkably, these 5 CpGs are located adjacent to the previously reported Nrf2 promoter [17]. Thus, the methylation status of these specific CpGs appears to be correlated with the tumorigenicity as well as Nrf2 expression and NQO1 induction (Figures 1 and 4). Notably, similar pattern of specific methylation of the distal CpG island has also been observed in the Keap1 gene in lung cancer cells [26]. It is important to note that some of our current findings appear to be somewhat contradictory with the results reported previously [11], however previous findings of extensive down-regulation of Nrf2 and GST during prostate tumor progression in TRAMP mice [11], are consistent with our current results.

To determine the functional role of methylation of these 5 CpGs in the suppression of Nrf2 expession, luciferase reporters of the Nrf2 promoter with or without these 5 CpGs were constructed (Figure 2A). The Nrf2 promoter possesses very GC-rich non-canonical promoter which contains neither TATA box nor a CCAAT box [28], however, this Nrf2-promoter potently activated the transcription of luciferase reporter gene (Figure 2B). Interestingly, the addition of sequences from −1065 to −1367 appears to be repressive to the transcriptional activity of the Nrf2 promoter (Figure 2B). Such repressive sequence could function by recruiting specific repressing factors [36], but the exact mechanism accounting for this repressive function of this sequence even in the absence of methylation would require further investigation. Nevertheless, when the reporters were methylated *in vitro* by CpG methyltransferase, the luciferase reporter activity of the Nrf2 promoter with the additional sequence containing the 5 CpGs (pGL-1367) was reduced by about 84%. In contrast, methylation of the reporter without the additional sequence resulted in about 23% reduction (Figure 2B). This is probably due to the heavy methylation of the whole construct including the luciferase gene (but further study would be needed to prove this). Altogether, these results suggest that the extra 5 CpGs (−1065 to −1367) could play a critical role in methylation-dependent suppression of Nrf2 promoter activity.

The role of CpG methylation in suppressing Nrf2 expression and activation was tested by treatment with 5-aza in TRAMP cells. 5-Aza has previously been shown to be able to prevent early disease progression, delay androgen-independent disease and improve survival of TRAMP mice [25], [37]. In addition, stage and phenotype-specific CpG island methylation and DNA methyltransferase expression have been well documented during prostate cancer progression in TRAMP mice [38], [39]. These published findings suggest the relevance of using this TRAMP system to interrogate the possible role of epigenetic alterations in prostate carcinogenesis. 5-aza treatment of TRAMP C1 cells modestly increased the mRNA level of Nrf2, while combined treatments of 5-aza and TSA induced a more prominent increase of Nrf2 (Figure 4A). This result is consistent with the report by Mavis et al., in which combined 5-aza and TSA treatments significantly enhanced the expression of GST genes [9]. The protein level of Nrf2 in TRAMP C1 cells remained unaffected by either 5-aza or 5-aza/TSA treatments, however, addition of a potent Nrf2-activator tBHQ would enhance the accumulation of Nrf2 protein (Figure 4B). As expected, the induction of NQO1 mRNA and protein levels displayed a similar trend with that of the Nrf2 protein. It is highly likely that since Nrf2 signaling is primarily regulated via post-translational mechanisms, without challenges with Nrf2-activators such as tBHQ, Nrf2 protein would be rapidly turned over by proteosome-dependent degradation [18]. The precise reason as to why tBHQ is needed for NQO1 induction would require further study.

To further delineate the molecular mechanism by which the specific CpG-methylation suppresses Nrf2 expression, ChIP assays were performed. The result reveals that the binding of MBD2 and H3K9m3 to the specific CpGs was substantially higher in TRAMP C1 cells than in TRAMP C3 cells correlating with the fact that the CpGs were minimally methylated in TRAMP C3 cells (Figure 3A). In contrast, AcH3 displayed an opposite binding pattern to the same CpGs sequence in TRAMP C1 cells as compared to TRAMP C3 cells, and similarly the binding of Pol II to the transcription start site showed similar pattern as AcH3 (Figure 3A). These methylation-dependent associations of corepressors could be modulated by 5-aza and TSA treatments and our results (Figure 3B) correlated very well with the transcription level of Nrf2 (mRNA level) in these two cell lines (Figure 4A). MBD2 has been reported to mediate epigenetic silencing of 14-3-3σ in TRAMP C1 cells and human LNCaP prostate cells [24], and has been shown to be involved in the transcriptional repression of GSTP1 in MCF-7 breast cancer cells [40]. MBD2 and other MBD proteins bind to methylated CpGs and recruit corepressor complexes which contain HDACs, chromatin remodeling proteins as well as other proteins leading to the repression of the expression of hypermethylated genes [31]. Interestingly, the protein level of MBD2 was much higher in TRAMP C1 cells than in TRAMP C3 cells (Figure 4A), suggesting a possible common MBD2-mediated epigenetic suppression of Nrf2 in TRAMP C1 cells. In contrast, the protein level of AcH3 was apparently lower in TRAMP C1 cells than in TRAMP C3 cells but was increased tremendously by TSA treatment (Figure 4D). Since the expression of MBD2 would be dependent on HDAC activities, our above results would potentially explain the requirement of HDAC inhibitors in order to effectively modulate corepressors binding and to maximally restore the reexpression of Nrf2 as well as induction of NQO1 in TRAMP C1 cells. In addition, when this suppressive sequence containing the extra 5 CpGs was further analyzed using the Transcriptional Elements Search System (TESS, http://www.cbil.upenn.edu/tess) based on the TRANSFAC V6.0 database, several transcription factor binding sites were identified, including the binding sites of E2F1-p107 and NF-E2 (data not shown). The exact binding proteins and their functions leading to the suppression of Nrf2 expression would require further investigation.

In summary, our present results clearly demonstrate that the expression of Nrf2 is suppressed by promoter CpG methylation in TRAMP prostate tumors. The existence and possible biological consequences of such epigenetic mechanism in the regulation of Nrf2 expression in human prostate cancer is currently under investigation in our laboratory. To the best of our knowledge, this is the first report revealing the epigenetic regulation of Nrf2 in prostate tumorigenesis. These findings would certainly open the door for further study on the role of Nrf2 as a plausible target for cancer chemoprevention and a possible diagnostic marker for detection of human prostate cancer.

## Materials and Methods

### Reagents and Cell Culture

All the enzymes used in the present study were obtained from New England Biolabs Inc. (Ipswich, MA) unless specified. Human recombinant insulin was purchased from Invitrogen. Dual luciferase assay system, luciferase reporter vectors pGL 4.75 with CMV promoter and pGL 4.15 were obtained from Promega (Madison, WI). All other chemicals were purchased from Sigma (St Louis, MO, USA).

TRAMP C1 and C3 cells were maintained in DMEM supplemented with 10% heat-inactivated fetal bovine serum, 5 µg/ml human recombinant insulin, 10^−8^ mol/L 5-androstan-17β-ol-3-one, and antibiotics. The cells were grown at 37°C in a humidified 5% CO_2_ atmosphere.

### Animals

Female hemizygous C57BL/TGN TRAMP mice, line PB Tag 8247NG, and male C57BL/6 mice were purchased from The Jackson Laboratory (Bar Harbor, ME). The animals were bred on same genetic background and maintained in the Animal Care Facility of Rutgers University. Housing and care of the animals were in accordance with the guidelines established by the University's Animal Research Committee consistent with the NIH Guidelines for the Care and Use of Laboratory Animals. Transgenic males used in the current study were routinely obtained as [TRAMP × C57BL/6] F1 or as [TRAMP × C57BL/6] F2 offspring. Identities of transgenic mice were confirmed by the PCR-based genotyping. Throughout the experiment the animals were housed in cages with wood chip bedding in a temperature-controlled room (68–72°F) with a 12-h light dark cycle, at a relative humidity of 45–55%, and fed with irradiated AIN-76A diet (DYETS Inc, Bethlehem, PA).

### Bisulfite Genomic Sequencing (BGS)

Genomic DNA was isolated from the palpable prostate tumors of 24 weeks old TRAMP mice (n = 12), apparently normal prostate tissue of 24 weeks old C57BL/6J mice (n = 10), TRAMP-C1 and C3 cells using the DNeasy tissue kit (Qiagen, Valencia, CA). The bisulfite conversion was carried out using 500 ng of genomic DNA with the use of EZ DNA Methylation Gold Kits following manufacturer's instructions (Zymo Research Corp., Orange, CA). The converted DNA was amplified by PCR using Platinum Blue PCR SuperMix (Invitrogen, Grand Island, NY) with specific primer sets (Table S1), with the translation initiation site (TIS) defined as position 1. The PCR products were purified by gel extraction using the Qiaquick™ gel extraction kit (Qiagen, Valencia, CA), then cloned into pCR4 TOPO vector using a TOPO™ TA Cloning kit (Invitrogen, Grand Island, NY). Plasmids DNA from at least 10 colonies per each group were prepared using QIAprep Spin Miniprep Kit (Qiagen, Valencia, CA) and sequenced (DNA Core Facility, Rutgers/UMDNJ, Piscataway, NJ).

### Plasmids

The genomic sequence of murine Nrf2 containing the promoter region was retrieved from NCBI mouse genome data base. Two murine Nrf2 promoter segments, −1065–1 and −1367–1 with the translation initiation site (TSS) referred to as position 1, were amplified from mouse genomic DNA isolated from normal mouse prostate using the following primers: 1065 forward, 5′-GGTACCTAAGTACGTGTAAAGGAACCCTGAGA-3′; 1367 forward, 5′-GGTACCAACAGTCACTACCACCACCA-3′; and a common reverse primer, 5′-CTCGAGGCTGAGGGCGGACGCTGT-3′. The PCR products were cloned into pCR4 TOPO vector using a TOPO TA Cloning kit (Invitrogen, Grand Island, NY) then digested with KpnI and XhoI and inserted into pGL4.15 luc2P/Hygro vector. All the sequences of recombinant plasmids were verified by sequencing (DNA Core Facility, Rutgers/UMDNJ, Piscataway, NJ).

The CpG-methylated reporters were generated by treating the reporter plasmids with methyl-transferase M. SssI according to the instruction provided by manufacturer. Briefly, 5 µg reporter constructs were incubated with 5 units of M. SssI for 1 hr in NEBuffer 2 (50 mM NaCl, 10 mM Tris-HCl pH 7.9, 10 mM MgCl_2_, and 1 mM dithiothreitol) supplemented with 160 µM S-adenosylmethionine at 37°C. Methylated plasmids were purified using QIAquick PCR purification kit (Qiagen, Valencia, CA) and the concentrations of all plasmids were determined by agarose gel electrophoresis. The efficiency of methylation reactions was confirmed by digestion using the methylation-dependent HhaI and HpaII restriction endonucleases (Figure S2).

### Transfection and Luciferase Reporter Assay

TRAMP C1 cells were plated in 24-well plates for 24 hrs, then transfected with 100 ng of the indicated reporter plasmids by using GeneJuice (Novagen, Madison, WI) according to the manufacturer's instructions. 25 ng of pGL 4.75, which contains a Renilla reniformis luciferase gene driven by CMV promoter, was co-transfected as internal control. 24 hrs after transfection, the cells were lysed in dual luciferase lysis buffer, and 10 µl aliquots of the cell lysate were assayed using a dual luciferase assay kit with a Sirius luminometer (Berthold Technologies, Pforzheim, Germany). The transcriptional activities of each constructs were calculated by normalizing the firefly luciferase activities with corresponding Renilla luciferase activities, and were reported as folds of induction compared with the activity of empty pGL 4.15 vector. The values are mean±SD of four separate samples.

### Cell Treatments and Western Blotting

Cells were plated in 6-well or 12-well plates for 24 hrs, then treated with 2 µM 5-aza, 200 nM TSA, or 1 µM 5-aza plus 100 nM TSA in media containing 0.5% FBS for 60 hrs, or 48 hrs followed by incubation in the presence of 5 µM tert-butylhydroquinone (tBHQ) for additional 12 hrs. After treatments, the cells were harvested in radioimmunoprecipitation assay (RIPA) buffer (Sigma, St Louis, MO). The protein concentrations of the cleared lysates were determined by using the bicinchoninic acid method (Pierce, Rockford, IL), and aliquots each containing 20 µg of total protein were resolved by 4%–15% SDS-polyacrylamide gel electrophoresis (Bio-rad, Hercules, CA). After electrophoresis, the proteins were electro-transferred to polyvinylidene difluoride (PVDF) membrane (Millipore, Bedford, MA). The PVDF membrane was blocked with 5% fat-free milk in phosphate-buffered saline-0.1% Tween 20 (PBST), then sequentially incubated with specified primary antibodies and HRP-conjugated secondary antibodies. The blots were visualized by SuperSignal enhanced chemiluminiscence (ECL) detection system and documented using a Gel Documentation 2000 system (Bio-Rad, Hercules, CA).

### RNA Isolation and Reverse Transcription-PCR

Total RNA was extracted from the treated cells using the Trizol (Invitrogen, Carlsbad, CA). Steady-state mRNA levels of Nrf2 and NQO1 were determined by semi-quantitative reverse transcription-PCR (RT-PCR). First-strand cDNA was synthesized from 2 µg of total RNA using SuperScript III First-Strand Synthesis System for RT-PCR (Invitrogen, Grand Island, NY) according to the manufacturer's instructions. The cDNA was used as the template for PCR reactions performed using Platinum Blue PCR SuperMix (Invitrogen, Grand Island, NY). The PCR products were isolated by agarose gel electrophoresis and visualized by EB staining using a Gel Documentation 2000 system (Bio-Rad, Hercules, CA). Primers to specifically amplify the genes involved were shown in Table S2.

### Chromatin Immunoprecipitation (ChIP) Assay

Chromoatin immunoprecipitation (ChIP) assay was carried out using Millipore's Magna-ChIP A kit (Millipore, Lake Placid, NY) following manufacturer's protocol. In brief, freshly prepared 18.5% formaldehyde was added to the cells (∼1×10^7^ cells in 150 mm dish) at a final concentration of 1%. Cells were incubated at 37°C for 10 min, then excess formaldehyde was quenched by addition of 5M glycine. After washing twice, the cells were scraped into 2 ml cold PBS containing 1× protease inhibitor cocktail II. The cells were pelleted and then resuspended in Cell Lysis Buffer containing 1× protease inhibitor cocktail II. Nuclei were isolated after Dounce homogenization and resuspended in Nuclear Lysis Buffer containing 1× protease inhibitor cocktail II. The samples were sonicated on ice using a Bioruptor sonicator (Diagenode Inc., Sparta, NJ) to shear the cross-linked DNA to an average length of 200–1000 bp and centrifuged at 12,000 rpm to remove insoluble material. The chromatin solutions were diluted 10-folds using dilution buffer, and 10 µl of each was reserved as total input control. Diluted chromatin solutions were precleared with salmon sperm DNA-protein A magnetic beads for 1 hr, and then incubated with protein A magnetic beads and antibodies specific for MBD2, Pol II, H3Ac and H3K9me3 (Millipore, Lake Placid, NY) or nonspecific IgG overnight at 4°C. The immunoprecipitated complex-magnetic beads were collected using magnetic separator and washed according to manufacturer's instruction. The pellets were then incubated with proteinase K in ChIP Elution buffer for 2 hrs at 62°C with shaking to elute immunocomplex and reverse cross-link. The samples were incubated at 95°C for 10 min and DNA was purified according to manufacturer's instruction. 1 µl of each of the purified DNA was used as template for 30 cycles of PCR amplification using designated primers (Table S2). The PCR products were then analyzed by agarose gel electrophoresis and visualized using EB staining. Primer set was designed to cover the DNA sequence from position −1190 to −1092 (mNrf2P1) in which the first 5 CpGs locate. To determine the association of RNA polymerase complex II to the Nrf2 promoter, another primer set was designed to cover the sequence closer to the transcription start site (TSS, −62 to +20, mNrf2P2). Primers covering β-actin promoter region was used as a control to verify the efficacy of ChIP assays.

## Supporting Information

Figure S1 Typical bisulfite genomic sequencing chromatographs show that the first 5 CpGs (indicated by arrows) are methylated in TRAMP prostate tumor but not in normal prostate. All the non-methylated cytosines were converted into thymidine by bisulfite treatment, while the methylated cytosines remained unchanged.
(0.22 MB PDF)

Figure S2 pGL-1065 and pGL-1367 reporters were methylated *in vitro* by CpG methyltransferase M. sssI, then digested by HpaI or HhaII. HpaI and HhaII are CpG-methylation-sensitive restriction endonucleases whose activity is blocked by CpG methylation.
(0.09 MB PDF)

Figure S3 pGL-1367 or pGL-1065 reporters, either methylated by CpG methyltransferase or not, were co-transfected with pGL 4.75 vector which contains a Renilla reniformis luciferase gene driven by CMV promoter into Hep G2 cells (A) or HEK293 cells (B), and the luciferase activities were measured after 24 hrs. The transcriptional activities of each constructs were calculated by normalizing the firefly luciferase activities with corresponding Renilla luciferase activities, and are represented as folds of induction compared with the activity of empty pGL 4.15 vector. The values are mean Â±SD of four separate samples.
(0.08 MB PDF)

Figure S4 The normalized expression of NQO1 in human prostate specimens is decreasing along with the progression of prostate cancer. Gene expression profiling data sets were retrieved from online database (www.oncomine.org) and analyzed for the expression of NQO1 in normal benign prostate, prostate carcinoma and metastatic prostate tumor.
(0.09 MB PDF)

Table S1 (0.03 MB DOC)

Table S2 (0.03 MB DOC)
