# Supplementary figures and images for: Nrf2 Expression Is Regulated by Epigenetic Mechanisms in Prostate Cancer of TRAMP Mice
Source: PLoS One. 2010 Jan 5;5(1):e8579. doi: 10.1371/journal.pone.0008579 (PMC2799519; doi:10.1371/journal.pone.0008579)

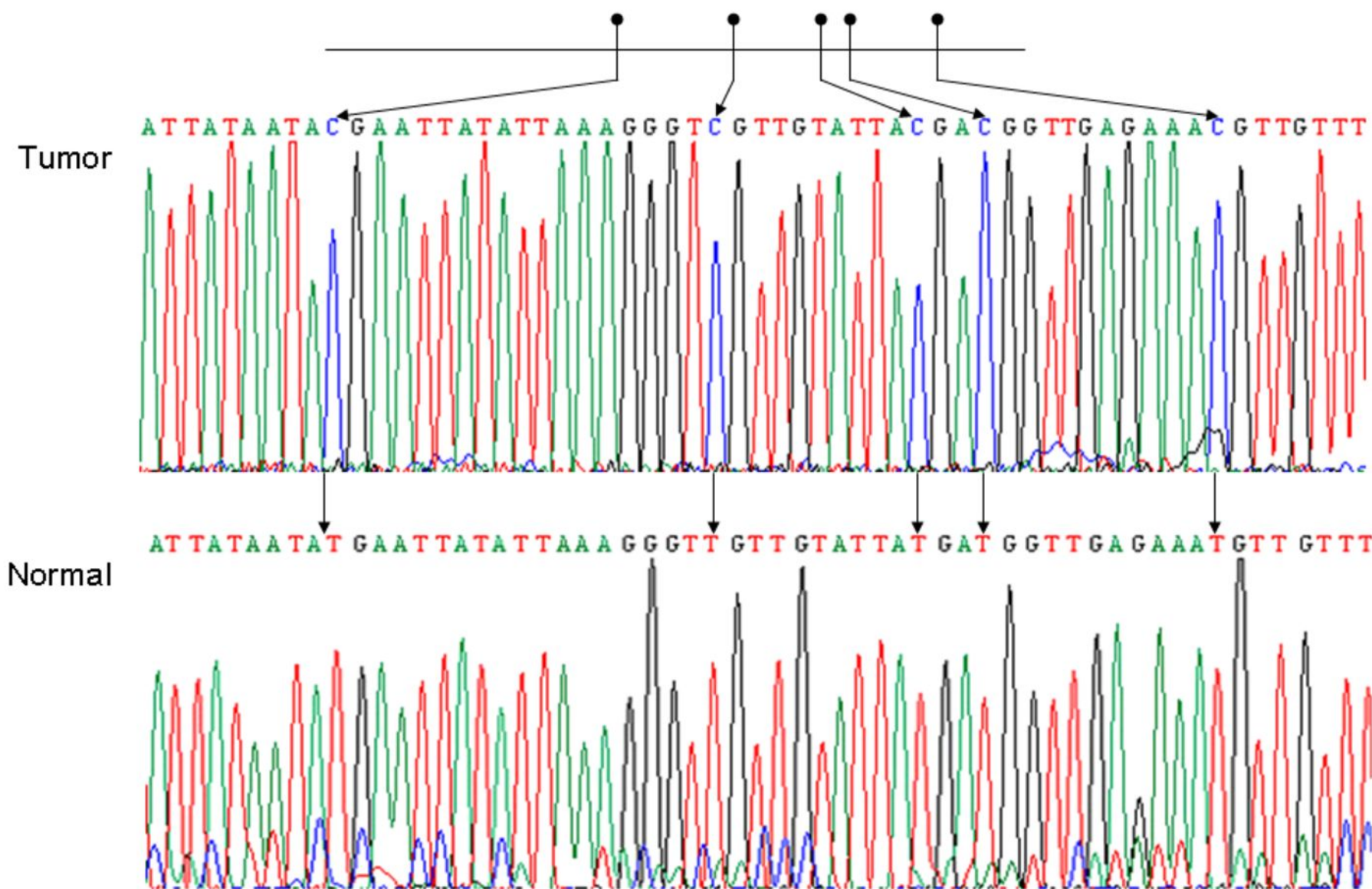

Supplementary figure 1

Supplement: Figure S1 — Typical bisulfite genomic sequencing chromatographs show that the first 5 CpGs (indicated by arrows) are methylated in TRAMP prostate tumor but not in normal prostate. All the non-methylated cytosines were converted into thymidine by bisulfite treatment, while the methylated cytosines remained unchanged. (0.22 MB PDF) [file pone.0008579.s001.pdf]

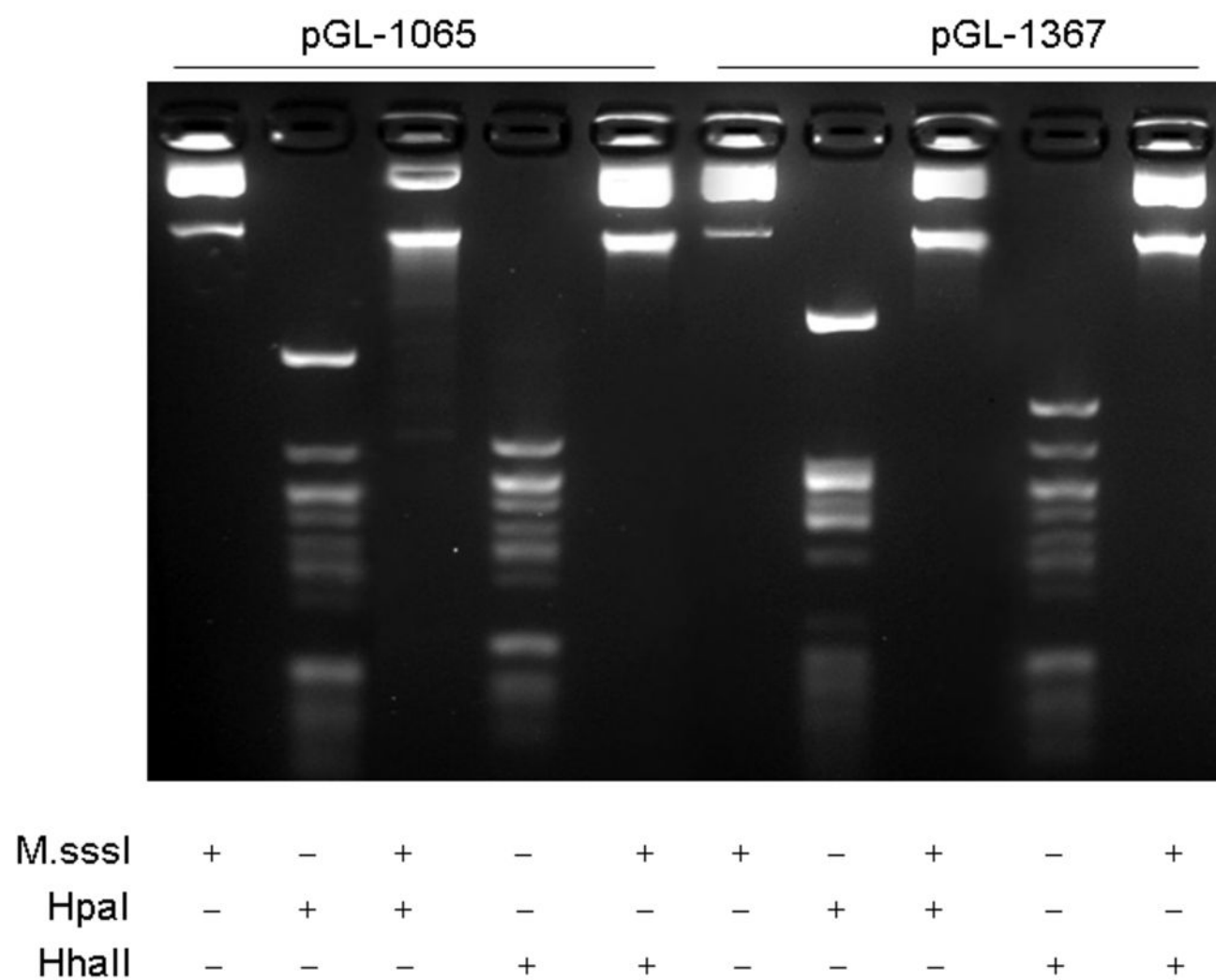

Supplementary figure 2

Supplement: Figure S2 — pGL-1065 and pGL-1367 reporters were methylated in vitro by CpG methyltransferase M. sssI, then digested by HpaI or HhaII. HpaI and HhaII are CpG-methylation-sensitive restriction endonucleases whose activity is blocked by CpG methylation. (0.09 MB PDF) [file pone.0008579.s002.pdf]

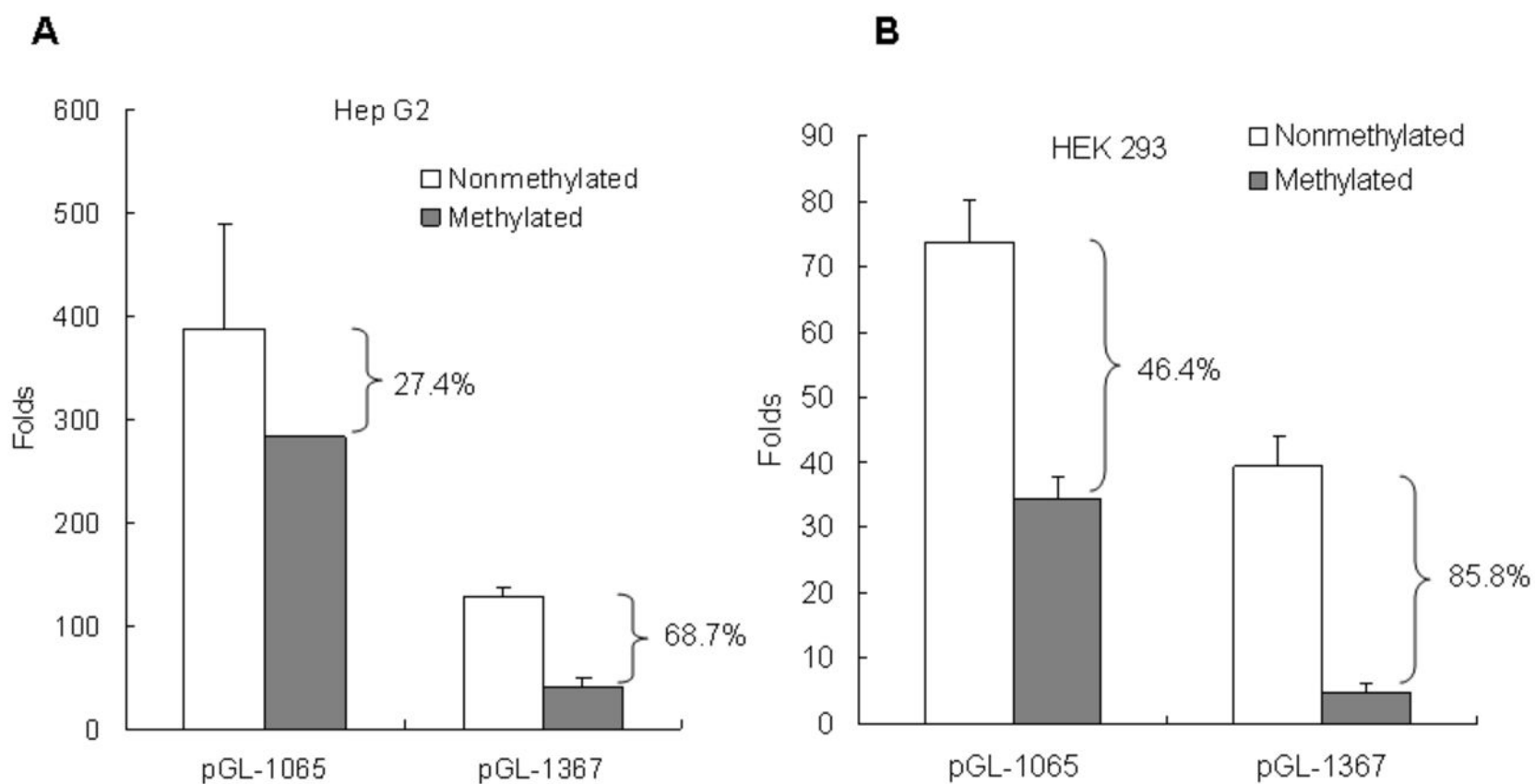

**Supplementary figure 3**

Supplement: Figure S3 — pGL-1367 or pGL-1065 reporters, either methylated by CpG methyltransferase or not, were co-transfected with pGL 4.75 vector which contains a Renilla reniformis luciferase gene driven by CMV promoter into Hep G2 cells (A) or HEK293 cells (B), and the luciferase activities were measured after 24 hrs. The transcriptional activities of each constructs were calculated by normalizing the firefly luciferase activities with corresponding Renilla luciferase activities, and are represented as folds of induction compared with the activity of empty pGL 4.15 vector. The values are mean Â±SD of four separate samples. (0.08 MB PDF) [file pone.0008579.s003.pdf]

NQO1  
NAD(P)H dehydrogenase, quinone 1

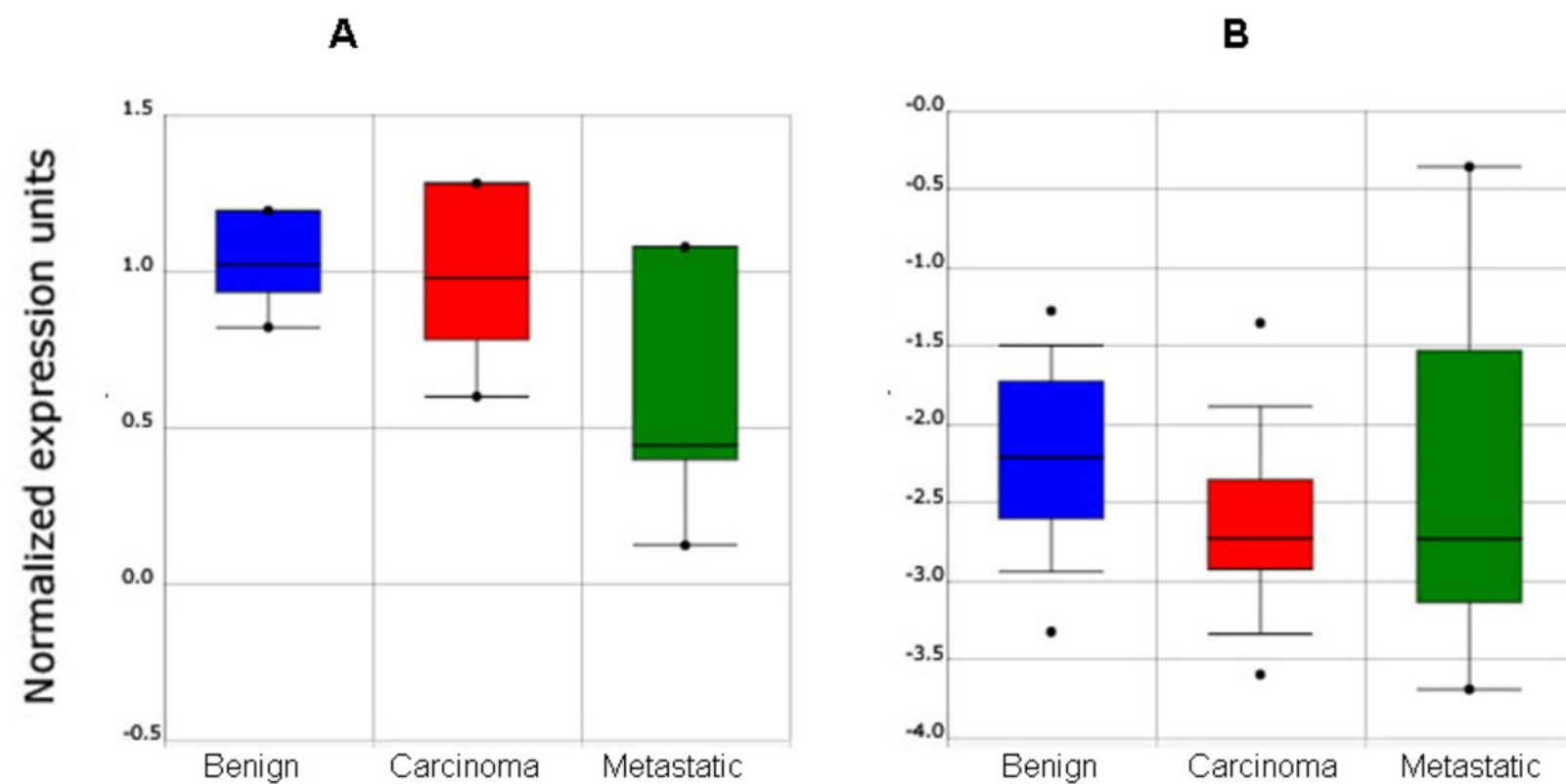

Supplementary figure 4

Supplement: Figure S4 — The normalized expression of NQO1 in human prostate specimens is decreasing along with the progression of prostate cancer. Gene expression profiling data sets were retrieved from online database (www.oncomine.org) and analyzed for the expression of NQO1 in normal benign prostate, prostate carcinoma and metastatic prostate tumor. (0.09 MB PDF) [file pone.0008579.s004.pdf]
