# Supplementary material for: Nrf2 Expression Is Regulated by Epigenetic Mechanisms in Prostate Cancer of TRAMP Mice
Source: PLoS One. 2010 Jan 5;5(1):e8579. doi: 10.1371/journal.pone.0008579 (PMC2799519; doi:10.1371/journal.pone.0008579)
Supplement: Table S1 — (0.03 MB DOC) [file pone.0008579.s005.doc]

Supplementary table 2. Primers used for RT-PCR and ChIP assay.

| Primer name | Sequence (5’ - 3’) | Product size |
| --- | --- | --- |
| mNrf2RT-F | GCT TTT GGC AGA GAC ATT CC | 242 bp |
| mNrf2RT-R | ATC AGC CAG CTG CTT GTT TT |
| mNQO1RT-F | CAG ATC CTG GAA GGA TGG AA | 596 bp |
| mNQO1RT-R | AAG TTA GTC CCT CGG CCA TT |
| mNrf2P1(-1190)-F1 | GAG GTC ACC ACA ACA CGA AC | 98 bp |
| mNrf2P1(-1092)-R1 | ATC TCA TAA GGC CCC ACC TC |
| mNrf2P2(-62)-F2 | CCT CAC CTC TGC TGC AAG TA | 82 bp |
| mNrf2P2(+20)-R2 | GGC AAC TCC AAG TCC ATC AT |
| ActinP-F | CCG GTC GAG TCG CGT CCA CC | 80 bp |
| ActinP-R | GGC GAA CTG GTG GCG GGT GT |
